# A 20-year retrospective study of osteoarticular tuberculosis in a pediatric third level referral center

**DOI:** 10.1186/s12890-021-01631-2

**Published:** 2021-08-16

**Authors:** Napoleón González Saldaña, Mercedes Macías Parra, Luis Xochihua Díaz, Martin Palavicini Rueda, Ana Jocelyn Carmona Vargas, José Iván Castillo Bejarano, Quetzalli Veloz Corona, Hugo Juárez Olguín, Juan Luis Chavez Pacheco

**Affiliations:** 1 Department of Infectious Diseases, Instituto Nacional de Pediatria (INP), Insurgentes Sur Avenue No. 3700-C, Cuicuilco District, CP 04530 Mexico City, Mexico; 2 Medical Director, Instituto Nacional de Pediatria , Mexico City, Mexico; 3 Department of Traumatology and Orthopedics, INP, Mexico City, Mexico; 4 Laboratorio de Farmacología, Instituto Nacional de Pediatría, Avenida Imán N° 1, 3rd piso Colonia Cuicuilco, CP 04530 Mexico City, Mexico; 5 Department of Pharmacology, Faculty of Medicine, Universidad Nacional Autónoma de Mexico, CP 04510 Mexico City, Mexico

**Keywords:** Children, Diagnosis of tuberculosis, Epidemiology, Osteoarticular tuberculosis

## Abstract

**Purpose:**

The objective of the present study is to describe the clinical, diagnostic, radiological and therapeutic aspects of osteoarticular tuberculosis (OATB) in patients in a tertiary pediatric hospital, to know if the diagnosis of OATB in pediatrics is a challenge due to its insidious clinical presentation.

**Methods:**

A retrospective, descriptive study of the cases of Tuberculosis (TB) in children was carried out. A total of 159 cases met the condition for the analysis.

**Results:**

The most frequent TB modality was extrapulmonary in 85%. Out of this, only 29% was OATB. The mean age was 4.9 years (range 8 months–16 years). Eighty-six per cent of cases received Bacille Calmette-Guérin (BCG) vaccination at birth. Median time of symptoms prior to diagnosis was 8 months. Microbiological confirmation was achieved only in five cases, with a high sensitivity to the antimicrobial treatment. *Mycobacterium bovis* BCG strain Tokio 172 was confirmed in three cases. Mortality rate was 0% during the time of study

**Conclusion:**

Our study describes the epidemiological characteristics of OATB cases in Mexican children. This data revealed a high prevalence of bone and joint TB infection. Pediatric OATB should be considered in cases with lytic bone lesions, fever and local pain. In countries with BCG immunization program, *M. bovis* should not be forgotten as an etiological agent. The low detection rate with one technique approach highlights the urgent need for more sensitive test to diagnose OATB in children.

## Introduction

Tuberculosis is a disease that has been in existence for centuries, with report of skeletal lesions in early Neolithic population [1]. The causative agent is the bacillus *Mycobacterium tuberculosis,* a strict aerobic microorganism, alcohol-acid resistant first described on 24th March, 1882 by Robert Koch.

TB has been a persistent global health problem with annual incidence rate of approximately 10 million cases, and one of the 10 leading causes of death in the world. In the last 5 years, it has been the leading cause of death by specific infectious agent, even above human immunodeficiency virus (HIV) [2].

The latest global report indicated that a total of 10.4 million peoples are infected and that out of these, 6% corresponds to children under 18 years of age. In the pediatric population, the most frequent site affected is the lung in 80%. Of the extrapulmonary presentations, 67% corresponds to lymph node TB, 13% to meningeal, 6% to pleural, 5% to miliary, and 4% to musculoskeletal manifestations [3]. Of the total cases of TB infection, osteoarticular involvement represents 4% to 5% with reports in the pediatric population of up to 7%. The diagnosis of extrapulmonary tuberculosis in the pediatric age is a challenge, since it presents with insidious onset without constitutional signs and symptoms in up to 72% m [4]. The objective of the present study is to describe the clinical, diagnostic, radiological and therapeutic aspects of OATB in patients in a tertiary pediatric hospital.

## Methods

This is a retrospective, descriptive study of the cases of Tuberculosis diagnosed in the Infectious Diseases Department of National Institute of Pediatrics, Mexico City from 1999 to 2018. The hospital has a unique program of Tuberculosis in Pediatrics and all the patients with TB diagnosis are directly managed by personnel who are specialists in the disease. We collected demographic data, BCG vaccination status, clinical presentation, imaging studies, performance of purified protein derivative (PPD), diagnostic time and method, medical-surgical treatment and follow up. Those patients who do not comply with previous criteria were excluded. HIV testing with ELISA was performed to detect immunodeficiencies. The operative procedure varied according to the case and comprises of arthrotomy and debridement, cryotherapy, application of bone substitutes (glass BONE™) and decompression with column stabilization. All methods were carried out in accordance with relevant guidelines and regulations under Ethics approval and consent to participate. As this was a retrospective study, all the information was obtained from the electronic records of the patients. However, the study was submitted to and approved by the Institutional Ethics and Academic Committee of National Institute of Pediatrics. Written informed consent was obtained from a parent or guardian for participants under 16 years old. The study was carried out with strict adherence to the ethical principles enunciated in the Declaration of Helsinki and to the CONSORT guidelines, http://www.consort-statement.org/consort-2010.

Two definitions of cases were used: (1) Confirmed case is defined as the presence of positive isolation of *Mycobacterium tuberculosis* complex in sputum; or in bone puncture fluid or tissue in a case with bone and joint infection; or a Ziehl–Neelsen (ZN) staining histologically compatible or positive; or Xpert MTB/RIF® positive and (2) probable case defined as the presence of 3 or more of the following criteria: (a) patient with consistent clinical data such as cough, fever and loss of weight, (b) compatible osteoarticular radiological image and/or Phemister’s triad (juxta-articular osteoporosis, peripheral bone erosion and decreased joint space), (c) positive contact, (d) PPD ≥ 10 mm and (e) favorable evolution with the onset of empirical anti-TB treatment. The conventional sites of affection were considered as (i) vertebral, (ii) hip, (iii) femur and (iv) knee.

### Statistical analysis

All data were statistically analyzed with SPSS system, version 21 (IBM). Descriptive statistics was used to report the characteristics of the population. The categorical variables were reported in percentages while the central tendency and dispersion measures, considered more appropriate, were used for the continuous variables. In addition, the X^2^ test was used. Values of *p* < 0.05 were taken to be statistically significant.

## Results

### Demographic characteristics

In the period from 1999 to 2018, 169 cases of Tuberculosis were diagnosed and out of this, 10 were excluded for incomplete information and follow-up. The presentation of TB was extrapulmonary in 144 cases (85%) out of which 43 (29%) presented involvement at osteoarticular level without significant difference in the gender distribution (boys 65% vs. girls 45%). The age range was from 8 months to 18 years with an average of 4.9 years. 35 (81.3%) of the cases occurred in children < 6 years old, 4 (9.3%) in those between 7 and 12 years and 5 (11.6%) in the patients between 13 and 16 years. In compliance with the national vaccination system of our country, 86% of the patients had a vaccination scheme with BCG applied at birth. 87% of the cases did not present comorbidities.

### Clinical presentation and location

Table 1 summarizes the clinical, microbiological and radiological characteristics of the patients. In patients with OATB; the most common symptoms were pain which was typically somatic and badly located in all the cases; fever in 37% of the cases (n = 16/43); asthenia-adynamia in 27% of the cases (n = 12/43) and edema in 9% (n = 4/43). All patients with Pott’s disease at the time of diagnosis presented a deformity of the spine cord. The average time of diagnosis of the cases was 8 months with a range of 1 to 48 months. Contact study was positive in 10 (23%) of the cases. The positive contact history did not decrease the time of the diagnosis (*p* < 0.05). Tuberculin test was performed in 11 cases (test sensitivity 45%). All patients were assessed in search of primary immunodeficiencies such as chronic granulomatous disease (CGD), interleukin 12-interferon gamma axis defect and infection by HIV, without finding any of these pathologies.

**Table 1:** Clinic and microbiological characteristics and sites of bone lesion

Clinical characteristics	n (%)
Mean duration of symptoms prior diagnosis	8 months (range 1–48 months)
Symptoms	n (%)
Pain	43 (100%)
Fever	16 (37%)
Loss of weight	2 (4%)
Edema of the joint	4 (9%)
Axial^a^ deformity	19 (44%)
Asthenia – adynamia	12 (27%)
Myalgia	3 (7%)
Headache	1 (2%)
Cough	1 (2%)
Abdominal pain	1 (2%)
*Microbiology*	
Culture ( +)^b^	5 (15%)
Ziehl–Neelsen staining ( +)^b^	5 (29%)
PCR ( +)^b^	5 (55%)
Histology ( +)	38 (97%)
PPD ( +)^b^	14 (46%)
Positive contact ( +)^b^	10 (23%)
*Sites of bone lesion*	
Thoracic column	14 (32%)
Lumbar column	4 (9%)
Hip	6 (14%)
Knee	5 (12%)
Costal grill	3 (7%)
Femur	2 (5%)
Talus	1 (2%)
Radius	1 (2%)
Tibia	1 (2%)
Maxilla	1 (2%)
Humerus	1 (2%)
Iliac	1 (2%)
Ankle	1 (2%)
Elbow	1 (2%)
*Surgical treatment*	
1. Curettage plus cryotherapy	30 (70%)
2. Arthrodesis	8 (19%)
3 Placement of glass BONE™	3 (7%)
4. Curettage with placement of cement	1 (2%)
*Medical treatment*	
1. Completed treatment	39 (90%)
2. Currently in therapy	3 (7%)
Median duration of completed treatment	13 months
1. Median duration of intensive phase	3.9 months
2. Median duration of maintenance phase	9.1 months
Mortality	0%

^a^The axial deformity was reported only for the cases of Pott’s disease

^b^Culture, Ziehl–Neelsen staining, PCR, PPD and positive contact study was performed in all the cases

The location of the infections was in conventional sites in 76% of the cases (n = 33/43). Column involvement occurred at thoracic level (T5–T9) in 32% of the cases (n = 14/43) followed by lumbar affectation in 9% (n = 4/43) and a case in the cervical level. All the cases of lumbar involvement presented concomitant dorsal affectation. The rest of the cases occurred in unusual sites. The sites of the TB cases were not related with age or sex of the patients (*p* > 0.05).

### Radiology

Chest radiographs were performed in all the cases with pathological data. According to imaging studies conducted in the sites of affectation, the most common findings were osteolytic lesion in all the cases and abscess in 70% of the cases. Phemister’s triad was observed in 9% of the cases (n = 4/43).

According to TC studies for Pott’s disease, the most common findings were osteolytic lesion, kyphosis and spinal cord compression in all the cases, while vertebral abscess occurred in 34% (n = 15/43).

### Diagnostic and microbiological studies

TB confirmation in the patients was performed by culture in 5 cases (sensitivity 15%), ZN staining in 5 cases (sensitivity 29%) and Polymerase chain reaction (PCR) in 9 cases (sensitivity 55%). Only one case had a positive culture and PCR results. The isolates reported in the culture were *M. tuberculosis* in sputum, *M. Tuberculosis* complex in 2 cases and 3 strains of *M. bovis* BCG Tokyo 172 (reported as adverse effect of the vaccine). Of the patients with negative culture, TB compatible biopsy with a sensitivity of 975 was observed in all cases. Only one case was defined as probable due to compliance with PDD ≥ 15 mm, isolation of *M. Tuberculosis* in sputum, imaging studies with compatible clinical data and also a favorable evolution with the onset of anti-TB treatment. We did not find reports of multi-drug resistant.

### Medical-surgical treatment

Thirty-nine patients (90%) completed the treatment while 3 (7%) are still with anti-TB therapy in maintenance phase and 1 (2%) patient, despite medical recommendation, abandoned the scheme after the intensive phase. First-line drugs (Isoniazid, Rifampicin, Ethambutol and Pyrazinamide) were used in all the cases. Clarithromycin was added to the anti-TB drugs during the intensive phase in only two of the patients while one patient conjunctively received quinolone with the anti-TB drugs in the maintenance phase. The addition of both drugs was due to the isolation of *Mycobacterium bovis,* strain BCG Tokyo 172 which is resistant to pyrazinamide. Of the patients who completed the treatment, the average duration of the intensive and maintenance phases were 3.9 months and 9.1 months respectively with total treatment duration of 13 months on average. In 7 patients, the treatment was prolonged (range 15 to 24 months) due to delay in the diagnostic tests to rule out immunodeficiency and a case of concomitant meningeal Tuberculosis.

Twenty-five patients required surgical treatment. In the cases of extraaxial involvement, curettage and cryotherapy were performed in 30 cases, anterior and posterior arthrodesis in 8 cases, curettage with placement of cement in 1 case and curettage with cryotherapy and placement of glass BONE™ in 3 cases. Patients with Pott’s disease were subjected to abscess drainage, debridement and stabilization. Mortality record was 0% during the study and only 2 adverse effects during the treatment were documented.

## Discussion

The diagnosis of OATB in pediatrics is a challenge due to its insidious clinical presentation. In this study, 43 cases of OATB handled under the TB unique program of the Infectious Diseases Department, National Institute of Pediatrics, Mexico City during a 20-year period were analyzed. Contrary to the national epidemiology where the pulmonary infection is the most common modality of tuberculosis, in our hospital, 85% of the cases were extrapulmonary presentations because lung TB are mostly managed in the second level of attention [5]. The presentation of OATB represented 29% of the general population, which is above the percentage in regions such as Europe, but coincides with what is reported in developing countries, particularly in Asia [6–8]. Patients ≤ 6 years old were the most prevalent due to the high risk of progression.

The most important clinical symptom was pain in all case followed by fever. Contact with TB diagnosis was identified in 23% of the patients; however, this finding did not modify the delay in the diagnostic time with an average of 8 months (in one case, the diagnostic delay was 2 years), unlike the report of Buonsenso et al. [9], where the patients with a history of positive contact had more timely diagnosis. However, the median time of pre-diagnostic symptoms in our study of 8 months coincides with the UK cohort reported by Kenyon PC et al., with a median duration of symptoms before diagnosis of 7 months and the Taiwan cohort reported by Chin-Yun et al., with a median time of 2 months [10, 11].

In the present study, TB location was not related to the age or gender of the patients. The localization of OATB was defined as conventional only in 24%,in comparison to the 42% reported in adult population by Jutte PC et al. [12]. Although this presentation might be associated to a delay diagnosis in children, more data is necessary to get a solid conclusion.

Bacteriological confirmation was poor with 85% and 71% negative culture and ZN staining respectively. The sensitivity of PPD in the present study was 46%, which was below the range of 50% to 90% in some studies of OATB [13, 14]. PCR test presented a sensitivity of 55% which is agreement with what was reported by WHO expert group [15]. The diagnostic method par excellence was histopathological study with a sensitivity of 97%. Of the 15% positive cultures, the presence of three *M. bovis* BCG Tokyo 172 strains calls for attention and represents the basis of a report on the adverse effect of the vaccine. This strain contains 30 million CFU/ml as compared to Pasteur 1173P3 sub-strain and Danesa 1331 both with 2 to 8 million CFU/ml; hence representing the highest concentration of this unit/ml. However, the strain presents less report of reactogenicity [16], and the presence of osteitis by BCG has a frequency of 1 case per million doses applied [17]. BCG osteitis cases have been described in countries with routine immunization programs. This finding highlights that *M. bovis* should be considered as an etiological agent in these countries. Further analysis in other countries is required to confirm this data.

All the patients in this study presented osteolytic lesions followed by abscess in 79% of the cases. However, the typical triad known as Phemister triad was only present in 9%, highlighting the importance of being on alert in the presence of unique osteolytic lesions. In patients with diagnosis of Pott’s disease, the use of simple radiographs may cause a delay of up to 19 months in the diagnosis; therefore, more advanced studies such as computer axial cosmography (CAT) magnetic resonance (RM) are extremely necessary [18]. In our population, the most common site of affectation was from T5 to T9 with the presence of osteolytic lesions, xiphosis and spinal cord compression in all the cases. Chest X-rays were also performed in search of concomitant pulmonary tuberculosis, finding alterations in 16% of the cases. However, it was confirmed only in two patients with sputum positive culture for *M. tuberculosis* which corresponds to 4% of the population and which was below the range of 6.9% to 29% reported in the literature [19, 20].

For the medical treatment, first-line anti-Tb drugs were used in all cases and antibiotics such as macrolides and quinolones were only added in three patients due to poor clinical evolution and isolation of *M bovis* BCG which is resistant to pyrazinamide [20]. The average total duration of treatment was 13 months which is in line with the recommendation of WHO [21]. During the treatment, only two adverse effects were reported. Evolution was favorable in all the cases and mortality rate was 0% and coincides with what was reported in other studies where the frequency of 0% to 16% was found [22].

We described a significantly low treatment abandonment of 2% of one patient after the intensive phase. According to a systematic review carried out by Kruk et al. [23], a lineal increase of 7% is estimated in the abandonment of treatment after the fourth week.

Surgical treatment in patients with vertebral involvement was necessary in 25% to 50% of the cases and in patients with diagnostic delay; this treatment may be as high as 98% [24, 25]. In the present study, all patients with Pott’s disease required surgical management with drainage of abscess, debridement and stabilization due to diagnostic delay which in these cases was 9 months on average. In the patients with extraaxial involvement, 60% underwent surgical procedures such as arthrodesis, curettage, cryotherapy and placement of bone substitution. A joint approach with the Immunology Department in search of primary immunodeficiency was undertaken without finding positive results; although, some reports described a frequency of concomitant presentation of HIV with osteoarticular TB of 10 to 27%, mainly in adult population [26].

## Conclusion

In conclusion, our study describes the epidemiological characteristics of OATB cases in Mexican children. This data revealed a high prevalence of bone and joint TB infection. In cases of lytic bone lesions, fever and local pain, pediatric OATB should be considered. In countries with BCG immunization program, *M. bovis* should not be forgotten as an etiological agent. The low detection rate with one technique approach highlights the urgent need for more sensitive test to diagnose OATB in children.

## Data Availability

All data generated or analyzed during this study are included in this published article. Besides, any additional data/files may be obtained from the corresponding author.
